# An Observational Study of Outcomes Associated With Virtual Pain Management Programs Based on Acceptance and Commitment Therapy Implemented During the COVID-19 Pandemic

**DOI:** 10.1097/AJP.0000000000001144

**Published:** 2023-07-04

**Authors:** Shakira Hollyfield, Warren Travers, Satwinder K. Sondh, Angelika Wilczek, Clair Jacobs, Lance M. McCracken, Whitney Scott

**Affiliations:** * INPUT Pain Management Unit, Guy’s and St Thomas’ NHS Foundation Trust; ‡ King’s College London, Health Psychology Section, Institute of Psychiatry, Psychology, and Neuroscience, London, UK; † Department of Psychology, Uppsala University, Uppsala, Sweden

**Keywords:** pain management, acceptance and commitment therapy, virtual delivery, chronic pain, pandemic

## Abstract

**Objective:**

In response to COVID-19, virtual, group-based interdisciplinary pain management programs (PMPs) were rapidly implemented. This included implementing different intensities and formats of virtual PMPs to address a range of patient needs and complexity. This observational study investigated outcomes associated with virtual high and low-intensity and pre-neuromodulation PMPs based on acceptance and commitment therapy as part of routine care during the pandemic.

**Methods:**

Depending on patients’ needs, participants completed a virtual high-intensity or low-intensity PMP, or a virtual PMP in preparation for neuromodulation, from June 2020 to June 2022. Participants completed standardized measures of pain intensity and interference, work and social adjustment, depression, and pain acceptance before and after treatment. Data from 2018 to 2019 for in-person residential (*n=*561), outpatient (*n*=123), and pre-neuromodulation (*n*=207) PMPs were also examined to provide a historical benchmark of performance.

**Results:**

The virtual high-intensity PMP (*n*=294) showed significant improvements in all variables, with small effects. There were significant improvements with small effects for pain interference, depression, and acceptance for the virtual pre-neuromodulation PMP (*n*=129). No statistically significant improvements were observed for the virtual low-intensity PMP (*n*=90). The improvements associated with prepandemic in-person PMPs were generally larger relative to the virtual PMPs of comparable intensity delivered during the pandemic.

**Discussion:**

These data provide preliminary support for the potential benefits of high, but not low, intensity virtual acceptance and commitment therapy-based PMPs, including in the context of neuromodulation. Research is needed to maximize the impact of virtual PMPs and match patients with the most appropriate delivery format.

## INTRODUCTION

Interdisciplinary pain management programs (PMPs) based on cognitive-behavioral therapy (CBT) are supported by meta-analyses of randomized-controlled trials (RCTs) and observational data.^1–5^ Acceptance and commitment therapy (ACT) is a more recent form of CBT for which evidence for persistent pain is growing.^6–9^ CBT and ACT-based pain management programs focus on helping people to manage the impact of persistent pain on their lives rather than on reducing pain itself. Consistent with this, research indicates that these approaches generally produce larger benefits in physical, social, and emotional functioning than in pain intensity.^5,7^

ACT is increasingly used within interdisciplinary PMPs in routine clinical practice.^10–12^ To address varying levels of patient need and complexity, different formats and intensities of ACT-based PMPs have been implemented within the UK’s National Health Service.^13^ For example, an intensive residential ACT-based PMP has been running for more than 12 years for patients presenting with severe and complex pain-related disability or distress.^14,15^ A less intensive outpatient format is also offered for people presenting with less pervasive impacts of pain on their lives.^16^ Finally, ACT-based PMPs have also been implemented for patients that are medically suitable for neuromodulation.^17^ ACT-based PMPs, in this context, help patients to make an informed decision about neuromodulation and to develop skills to respond more effectively to pain, irrespective of the outcome of neuromodulation.^17^

The COVID-19 pandemic caused an unprecedented impact on health care delivery worldwide.^18^ Within this context, there was a need to rapidly adapt interdisciplinary PMPs for remote delivery to reduce service disruption. Increasing evidence from RCTs of Internet-delivered CBT for pain, including ACT, support the potential benefits of remote delivery.^19–22^ In addition, evidence from 1 noninferiority trial suggests that in-person and remotely-delivered ACT for pain produce comparable outcomes.^23^ Before the pandemic, however, remotely-delivered PMPs were not widely implemented, with notable exceptions.^24^ Research is therefore needed to understand the effectiveness of remotely-delivered PMPs implemented in real-world practice where patients present with greater complexity.^24^ Crucially, implementation must consider the local context, including permitted technological platforms, staff resources, and support to sustainably deliver and continuously improve remote treatment.^25–27^

Remote delivery of PMPs is not without challenges. Key among these is maintaining patient engagement.^18^ Difficulties fostering a therapeutic alliance, privacy and security concerns, and poor digital literacy are potential challenges to address to optimize inclusion and engagement in this format.^18,28^ Additionally, in-person PMPs are often delivered in a group, and processes such as group cohesion are thought to impact outcomes.^29^ However, Internet-based treatments for pain are typically delivered individually. There is also a need to understand outcomes associated with varying intensities and formats of remote PMPs to improve understanding of how to best address the varying complexity of patient needs.

This study, therefore, investigated outcomes associated with ACT-based PMPs delivered for groups through video platforms (“virtual PMPs”) in a specialty pain service during the pandemic. Before the pandemic, this service provided a range of in-person group-based PMPs to address varying needs including, as mentioned, intensive residential,^15^ outpatient,^16^ and neuromodulation preparation PMPs.^17^ Virtual PMPs mirroring the content and intensity of these in-person programs were developed during the pandemic. Thus, the service adapted each of the PMPs that were delivered in-person before the pandemic into a virtual format to support a range of patient needs during the pandemic. It was hypothesized that each of the 3 formats of virtual ACT-based PMPs would be associated with significant improvements in pain interference, work and social adjustment, depression, and pain acceptance. Given that the virtual PMP formats varied as a way to address differing patient needs, we did not make specific hypotheses about the relative performance of the different treatment formats, nor was it our intention to directly compare these. Nonetheless, data from the virtual high and low-intensity and pre-neuromodulation programs are presented together in this manuscript to illustrate implementation in practice and to examine the potential generality of results across treatment formats in the unique historical context of the pandemic. Although direct comparisons require cautious interpretation, prepandemic data from the in-person PMPs in this service are presented as a benchmark of previous performance.

## MATERIALS AND METHODS

### Participants

**FIGURE 1:**
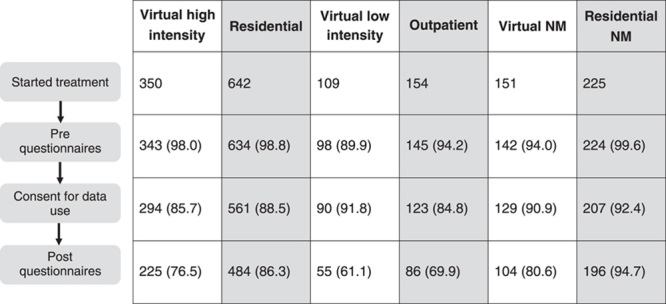
Participant flow diagram across treatment programs. NM indicates pre-neuromodulation.

This paper presents data from consecutive participants completing virtual PMPs during the COVID-19 pandemic from June 2020 to June 2022. This includes data from participants completing virtual high and low-intensity and pre-neuromodulation programs during this time. Data from consecutive participants completing in-person PMPs before the pandemic were also included (January 2018 to December 2019). This includes data from participants completing residential, outpatient, and pre-neuromodulation programs. Figure 1 shows the data collection process and the number of participants providing pretreatment and posttreatment data for each treatment program. Portions of the prepandemic residential PMP data have been previously published;^14^ however, outcome data from the other programs before and during the pandemic have not been published.

All participants were assessed by a clinical psychologist and advanced practitioner physiotherapist in pain management to determine the suitability of a group ACT-based PMP. Across all treatment formats, the general inclusion criteria were as follows: (1) being 18 years of age or older; (2) the presence of pain for 3 months or more, which significantly impacted daily function, mood, and/or overall quality of life, as judged by the assessing clinicians; and (3) willingness to attend and participate in a treatment that focused on improving quality of life and personal goals rather than on pain reduction. For the pre-neuromodulation programs, participants were willing to engage in treatment focused on these aims alongside learning technical information to make an informed decision about receiving neuromodulation, typically a spinal cord stimulator. The general exclusion criteria across treatment formats were as follows: (1) significant ongoing medical treatments, investigations, or procedures (with the exception of neuromodulation); (2) serious and poorly controlled psychiatric conditions (eg, active psychosis, severe posttraumatic stress disorder, active suicidality); (3) inability to engage constructively in group treatment, such as due to interpersonal difficulties or cognitive impairments; and (4) the use of liquid opioid medication while attending the program (immediate release tablets below 200 mg daily dose of morphine equivalence was acceptable).

There were additional inclusion and exclusion criteria depending on the specific delivery format and treatment pathway. These are presented in Table 1 for comparison. Clinicians’ judgment, based on the assessment information, in combination with consideration of patient preferences, informed the recommendation for the specific PMP format. The frequency of specific reasons for exclusion/attrition was not systematically recorded for this study. However, an audit (separate from the current study) conducted within the same service identified that the most common reasons for exclusion from the programs offered were the presence of serious and poorly controlled psychiatric conditions, not being ready for a self-management approach, and pain not significantly impacting on daily functioning.^13^

**TABLE 1:** Additional Inclusion and Exclusion Criteria According to Treatment Format

Specific criteria for in-person Programs (2018–2019)	
Residential	Ability to manage all aspects of their own self-care and manage the physical requirements necessary to attend and participate in the program while staying on site.
Outpatient	Ability to manage the physical requirements necessary to commute twice per week to and from the hospital and still actively participate in the program. Patients recommended for outpatient PMP generally have a higher level of physical/emotional/social functioning, warranting less intensive treatment as compared with those recommended for the residential program.
Residential pre-neuromodulation	Deemed medically suitable for neuromodulation after review by a neuromodulation consultation (eg, has a condition such as complex regional pain syndrome or localized neuropathic signs and symptoms). Ability to manage all aspects of their own self-care and manage the physical requirements necessary to attend and participate in the program while staying on site.
Specific criteria for virtual programs (2020-2022)	
High-intensity virtual	Suitable computing facilities (ie, a tablet, laptop, or desktop computer)^*^, Wi-Fi access, and private space with which to engage in the virtual program.
Low-intensity virtual	Suitable computing facilities (as above)^*^, Wi-Fi access, and private space with which to engage in the virtual program Patients recommended for the low-intensity virtual PMP generally have a higher level of physical/emotional/social functioning warranting less intensive treatment as compared with those recommended for the high-intensity virtual program.
Virtual pre-neuromodulation	Deemed medically suitable for neuromodulation (as above) Suitable computing facilities (as above)^*^, Wi-Fi access, and private space with which to engage in the virtual program

* From December 2020, the option of being loaned a tablet was available to participants so they could attend if they had Wi-Fi access but no suitable device.

### Procedure

All treatment participants were asked to complete a standardized and validated set of self-report measures at the start and end of their treatment program. The pretreatment measures gathered demographic information, including sex, age, ethnicity, pain location and duration, home situation, highest level of education, and work status. At pretreatment, participants completing virtual programs during the pandemic also responded to questions about whether they had experienced specific events related to COVID-19 and how the pandemic had affected their health care use, health, functioning, mood, pain, and social support. These data were collected for descriptive purposes to contextualize the outcome data. At both pretreatment and posttreatment, participants completed standardized measures of pain outcomes, namely, pain intensity, pain interference, work and social adjustment, and depressive symptoms. They also completed a measure of pain acceptance, a key treatment process variable at pretreatment and posttreatment. In addition, at posttreatment, participants provided ratings of their overall impression of change during treatment. All measures are described in detail below. Written informed consent was obtained from participants to use their data for research purposes.

Participants doing a prepandemic in-person program completed standard paper-based assessment measures in the clinic. Participants completing a virtual program were sent an email with a link to complete questionnaires online through ‘Online Surveys’ (https://www.onlinesurveys.ac.uk). Trained service staff were on hand to provide support and answer questions for participants completing questionnaires in the clinic and remotely. This process facilitated data completeness. This study was approved by the National Research Ethics Service Committee South Central – Oxford C (17/SC/0537) and was conducted in line with the Declaration of Helsinki.

### Treatment Approach Applied Across Delivery Formats

Before the pandemic, patients completed either a 3-week residential PMP, an outpatient PMP, or a 2-week residential pre-neuromodulation PMP depending on their needs, as discussed in the inclusion/exclusion criteria. During the pandemic, participants completed a virtual high or low-intensity program or a virtual pre-neuromodulation program; these were developed to correspond to the content and intensity of the residential, outpatient, and pre-neuromodulation programs, respectively. All treatment programs and delivery formats were based on the ACT model and focused on enhancing psychological flexibility.^7,30^ Program types and delivery formats, including clinicians involved and contact time, are summarized in Table 2.

**TABLE 2:** Details of Format and Delivery Across the In-Person and Virtual Programs

Type of pain management program	Treatment days	Treatment duration	Hours of contact	Clinical staff involved
Virtual high-intensity	12 d	3 wk	36 ^*^12^†^	φ, P, OT & N
Residential	15 d	3 wk	75	φ, P, OT & N
Virtual low-intensity	5 d	3 wk (4 wk from the end of 2021)	12.5 (20 from the end of 2021)^‡^	φ & P
Outpatient	5 d	3 wk	20	φ & P
Virtual pre-neuromodulation	8 d	2 wk	27 ^*^4^†^	φ, P, OT & N
Residential pre-neuromodulation	8 d	2 wk	40	φ, P, OT & N

* Signifies additional hours available for patients to meet and talk with other participants in the group online without clinician input.

† Signifies twice weekly 1:1 telephone calls for therapeutic input with patients by a named clinician.

‡ Signifies once weekly 1:1 telephone calls for therapeutic input with patients by a named clinician.

φ indicates clinical psychologist; P, advanced practitioner physiotherapist in pain management; OT, specialist occupational therapist; N, specialist pain nurse.

Across all program formats, the treatment approach included the use of metaphors, experiential exercises, mindfulness practice (including mindful movement exercises), value clarifications, values-based goal settings, and opportunities for practice and rehearsal. Treatment was aimed at broadening the behavioral strategies employed and increasing awareness of options for pursuing values-based actions. Throughout the program, clinicians applied ACT principles to highlight avoidance and applied goal achievement as a guide for action. ACT strategies helped participants become more aware of and respond more openly to challenging experiences (eg, pain, fear, shame, guilt, anger, sadness) and to pursue their values. All clinicians received regular training and updates on pain management practices and ACT through regular clinical development meetings. Psychoeducation on pain medication was also provided.

For programs where nurses were involved (Table 2), one-to-one telephone contact was made by a nurse to discuss an individual’s pain medication consumption and explore goals for pain medication reduction. Psychoeducation on pain physiology, anatomy, and allostatic loading and stress models were also applied flexibly as required. Additional home exercises, mindfulness practice, and weekend goals were discussed during the treatment period. Regular interdisciplinary team meetings to reflect, plan, and formulate formed an integral part of the team’s treatment strategy.

### Adapting Treatment for Virtual Delivery

The virtual programs were delivered on a video platform called BlueJeans, which was approved for use by the hospital. Participants were offered an appointment before the program to ensure that they were able to use the BlueJeans platform successfully. To maximize focus and engagement, the virtual treatments had regular breaks and employed group discussions, breakout rooms, multimedia resources (eg, short videos, audio recordings), and screen displays. Due to concerns that patients would have difficulty maintaining engagement and concentration 5 days per week in the intensive virtual treatment program, the overall number of days for this program was reduced to 12 compared with the 15 days of the residential format. The number of days for the virtual low-intensity and virtual pre-neuromodulation programs was consistent with their in-person counterparts.

To foster group cohesion and openness in the virtual high-intensity and pre-neuromodulation PMPs, an additional optional hour-long daily session was scheduled for patients to meet and talk with each other virtually without clinician involvement. This was not available in the virtual low-intensity program, but patients were encouraged to connect with each other outside of the formal program hours if they were able to. To develop the therapeutic alliance and mitigate challenges arising during virtual treatment, each patient was allocated to a named clinician who followed them up by phone. For the virtual high-intensity and pre-neuromodulation programs, patients were contacted by their allocated clinician a minimum of 2 times per week, while patients completing the virtual low-intensity PMP were contacted by their clinician once per week on average. The implementation of these features was shaped in response to patient feedback. There were generally 8 to 10 participants per group in the virtual treatments.

Following the initial implementation of the virtual low-intensity program, patient and clinician feedbacks indicated that the duration was insufficient to deliver sufficient content. Therefore, this program was increased from 12.5 to 20 hours at the end of 2021. Due to the small numbers, it was not possible to compare outcomes associated with the 12.5- and 20-hour versions of this program. Therefore, outcomes for patients completing either version of the virtual low-intensity program were combined for analysis.

### Assessment Measures

#### Pain Intensity

Participants rated their average pain intensity over the past week using a standard 11-point numerical rating scale with the end points 0 (no pain) to 10 (worst possible pain).

#### Brief Pain Inventory-Interference Subscale (BPI-IS)

The BPI-IS was used to measure the impact of pain on daily functioning in 7 domains: general activity, mood, walking ability, work (including housework), relationships with others, sleep, and enjoyment of life. Responses apply to the past week and require participants to rate the 7 items on an 11-point scale, ranging from 0 (does not interfere) to 10 (completely interferes).^31^ BPI-IS average scores were used, with higher average scores reflecting greater pain-related interference. The BPI-IS is a widely used outcome measure in chronic pain studies and is considered a reliable and valid measure for assessing pain-related interference with daily functioning.^32–34^

#### Work and Social Adjustment Scale (WSAS)

The WSAS was used to measure functional impairment associated with the participants’ health condition in 5 domains: work, home management, social leisure, and private leisure activities, and personal or familial relationships^35^ Participants rated the 5 items on an 8-point scale, ranging from 0 (no impairment) to 8 (very severe impairment). Higher scores indicate more severe impairment in work and social functioning. The WSAS is considered a reliable and valid measure for assessing functioning in people with long-term health conditions.^35,36^

#### Patient Health Questionnaire (PHQ-9)

The PHQ-9^37^ was used to assess participants’ depressive symptom severity, as defined by the standard diagnostic criteria for depression. Participants rated the frequency with which they experienced 9 symptoms of depression over the past 2 weeks on a 4-point scale, ranging from 0 (not at all) to 3 (nearly every day). Higher scores indicate more severe depression symptoms. The PHQ-9 is considered a reliable measure for assessing the severity of depression symptoms and has been validated among patients with a broad range of physical health conditions, including chronic pain.^38^

#### Chronic Pain Acceptance Questionnaire (CPAQ-8)

The 8-item version of the CPAQ was used to measure participants’ pain acceptance.^39^ The CPAQ consists of items related to participation in valued activities in the presence of pain and refraining from unsuccessful attempts to control, avoid, or reduce pain.^40^ Participants rated each item on a 7-point numerical scale ranging from 0 (never true) to 6 (always true). CPAQ-8 total scores were used, with higher scores indicating greater pain acceptance. There is evidence for the reliability and validity of the CPAQ-8, and it has shown good convergent validity with the original 20-item version of the CPAQ.^40,41^

#### Patient Global Impression of Change (PGIC)

At posttreatment, the PGIC was used to measure participants’ overall impression of change over the course of treatment.^42^ Participants rated their overall improvement following the general stem “Compared to how you were before treatment, how are you doing overall” on the following 7-point scale: 1 (very much improved), 2 (much improved), 3 (minimally improved), 4 (no change), 5 (minimally worse), 6 (much worse), and 7 (very much worse). The PGIC has been widely used in clinical trials of analgesic medications for chronic pain^43^ and has been shown to capture change related to a number of important outcome domains following the completion of an ACT-based PMP.^44^ The following anchors were used to describe ‘Meaningful improvement’ (1—very much improved, 2—much improved), ‘No meaningful change (3—minimally improved, 4—no change, 5—minimally worse), and ‘Meaningful worsening’ (6—much worse, 7—very much worse).

### Statistical Analysis

Preliminary statistical analyses were carried out using SPSS version 27 (SPSS Inc., Chicago, IL). Descriptive statistics were computed for all variables. Skewness, kurtosis, histograms, and Q-Q plots were examined for each variable to determine normality. Means and SDs were computed for continuous variables for descriptive purposes, and frequencies and percentages were computed for categorical variables. Participants who did not complete posttreatment measures were compared with those who did on all demographic and outcome/process variables, using independent samples *t*-tests for continuous variables and χ^2^ tests for categorical variables.

To examine change in the variables from pretreatment to posttreatment, intention-to-treat linear mixed models were run using restricted maximum likelihood estimation to maximize the use of all available data and account for the repeated measures nature of the data. The mixed models included a fixed effect of time and a random intercept. The analyses were run with the Jamovi Gamlj module. Cohen’s *d* was computed using the adjusted mean change from each mixed model divided by the pooled observed SDs. The following benchmarks for interpreting *d* were used: small (≥0.20), medium (≥0.50), and large (≥0.80).^45^ As a sensitivity analysis, missing posttreatment data were imputed using the baseline observation carried forward (BOCF), and the linear mixed models and effect sizes were re-computed. Frequencies and proportions were computed for the PGIC categories.

## RESULTS

### Sample Demographics and Experience of COVID-Related Events

Descriptive statistics for participants’ demographics according to the treatment program are summarized in Table 3. Briefly, across all programs, the samples were comprised predominantly of women and white participants. The mean age of participants ranged from 45.26 (SD=13.17) in the virtual low-intensity program to 50.16 (11.92) in the residential pre-neuromodulation program. Across the programs, participants had pain of longstanding duration (all means/medians >9 y). Low back pain was the most frequently reported primary pain location across the programs.

**TABLE 3:** Demographic Characteristics for All Participants Who Started Treatment Across Treatment Programs Before and During COVID-19

	Virtual high-intensity M(SD) or *n*(%)	Residential M(SD) or *n*(%)	Virtual low-intensity M(SD) or *n*(%)	Outpatient M(SD) or *n*(%)	Virtual NM M(SD) or *n*(%)	Residential NM M(SD) or *n*(%)
	Pretreatment n=294	Pretreatment n=561	Pretreatment n=90	Pretreatment n=123	Pretreatment n=129	Pretreatment n=207
Sex						
Women	238 (80.9)	437 (77.9)	71 (78.8)	95 (77.2)	67 (52.0)	121 (58.5)
Men	54 (18.4)	121 (21.6)	19 (21.2)	28 (22.8)	62 (48.1)	86 (41.5)
Missing	2 (0.7)	3 (0.5)	0 (0)	0 (0)	0 (0)	0 (0)
Age	47.21 (12.35)	48.12 (12.87)	45.26 (13.17)	45.66 (12.65)	49.18 (11.98)	50.16 (11.92)
Ethnicity						
Asian	23 (7.8)	40 (7.1)	5 (5.6)	8 (6.5)	5 (3.9)	1 (0.5)
Black	50 (17.0)	67 (11.9)	13 (14.4)	13 (10.6)	3 (2.3)	2 (1.0)
Mixed	13 (4.4)	26 (4.6)	5 (5.6)	7 (5.7)	4 (3.1)	5 (2.4)
Other	10 (3.4)	11 (2.0)	5 (5.6)	8 (6.5)	3 (2.3)	0 (0)
White	198 (67.3)	404 (72.0)	61 (67.8)	85 (69.1)	114 (88.4)	196 (94.7)
Missing	0 (0)	13 (2.3)	1 (1.1)	2 (1.6)	0 (0)	3 (1.4)
Education (y)	—	13.50 (3.58)	—	16.00 (5.00)^*^	—	12.00 (4.00)^*^
Primary	16 (5.4)	—	2 (2.2)	—	8 (6.2)	—
O-levels/GCSEs	80 (27.2)	—	22 (24.4)	—	55 (42.6)	—
A-levels	68 (23.1)	—	17 (18.9)	—	27 (20.9)	—
Uni. Bachelor’s	63 (21.4)	—	20 (22.2)	—	17 (13.2)	—
Uni. Post-graduate	41 (13.9)	—	21 (23.3)	—	10 (7.8)	—
Doctoral degree	1 (0.3)	—	3 (3.3)	—	1 (0.8)	—
Other	25 (8.5)	—	5 (5.6)	—	11 (8.5)	—
Employed						
Unemployed	174 (59.1)	308 (54.9)	12 (13.3)	28 (22.8)	69 (53.5)	90 (43.5)
Part/Full-time	79 (26.8)	142 (25.3)	64 (71.1)	70 (56.9)	38 (29.5)	66 (31.9)
Volunteer	1 (0.3)	8 (1.4)	0 (0)	5 (4.0)	1 (0.8)	4 (1.9)
Carer	1 (0.3)	3 (0.5)	1 (1.1)	3 (2.4)	0 (0)	3 (1.4)
Homemaker	3 (1.0)	14 (2.5)	1 (1.1)	0 (0)	0 (0)	7 (3.4)
Student	4 (1.3)	9 (1.6)	3 (3.3)	4 (3.3)	0 (0)	0 (0)
Retired	32 (10.9)	60 (10.7)	9 (10.0)	9 (7.3)	21 (16.3)	29 (14.0)
Missing	0 (0)	17 (3.0)	0 (0)	4 (3.3)	0 (0)	8 (3.9)
Pain duration (y)	14.06 (10.65)	10.23 (13.24)^*^	11.00 (12.02)^*^	9.01 (11.34)^*^	12.03 (8.98)	9.01 (14.52)^*^
Main pain						
Back	129 (43.9)	211 (37.6)	36 (40.0)	57 (46.3)	56 (43.4)	113 (54.6)
Widespread	40 (13.6)	102 (18.2)	8 (8.8)	11 (8.9)	4 (3.1)	4 (1.9)
Lower limbs	41 (13.9)	65 (11.9)	10 (11.1)	18 (14.6)	36 (27.9)	46 (22.2)
Upper limbs	23 (7.8)	30 (5.3)	16 (17.7)	12 (9.7)	12 (9.3)	10 (4.8)
Neck	22 (7.5)	32 (5.7)	5 (5.5)	7 (5.7)	2 (1.5)	5 (2.4)
Head/face	6 (2.0)	23 (4.1)	2 (2.2)	4 (3.2)	3 (2.3)	6 (2.9)
Abdominal	9 (3.1)	20 (3.6)	4 (4.4)	4 (3.2)	5 (3.9)	4 (1.9)
Pelvic	24 (8.2)	14 (2.5)	8 (8.8)	3 (2.4)	7 (5.4)	6 (2.9)
Anal/genital	0 (0)	3 (0.5)	0 (0)	0 (0)	4 (3.1)	4 (1.9)
Chest	0 (0)	6 (1.1)	1 (1.1)	3 (2.4)	0 (0)	0 (0)
Missing	0 (0)	55 (9.8)	0 (0)	4 (3.9)	0 (0)	9 (4.3)

Education was assessed in years from 2018 to 2019. From 2020 onwards, this was assessed as the highest level of education; NM, pre-neuromodulation.

* Median and interquartile range.

GCSEs indciates General Certificate of Secondary Education.

Across the virtual programs, the majority of participants had not experienced a significant COVID-related event (Supplementary Table 1, Supplemental Digital Content 1, http://links.lww.com/CJP/A995). However, a minority of participants experienced events such as losing their job, the death of a family member or friend, a major financial change for the worse, and/or a change in their living situation. Across all virtual programs, 27-41% of participants reported meaningful worsening in their health/functioning overall, and 29% to 56% reported worsening in the areas of physical activity, work, mood, social activities, and pain intensity due to the COVID-19 pandemic.

### Pretreatment Differences Between People Who Did And Did Not Complete Posttreatment Questionnaires

Participants who completed posttreatment questionnaires did not differ significantly from those who did not complete these on any baseline variable across all 3 virtual programs. Within the 3-week residential program and the pre-neuromodulation residential program, participants who completed posttreatment questionnaires did not differ significantly from those who did not complete these on any baseline variable. For the in-person outpatient program, participants who completed posttreatment questionnaires were significantly younger (M=43.74, SD=11.91) than those who did not complete these (M=49.86, SD=13.37), *t*(106)=2.38, *P*<0.05. Outpatient program participants who completed questionnaires also had significantly lower pretreatment pain interference (M=6.18, SD=1.97) than those who did not (M=6.87, SD=1.62), *t*(78.90)=2.00, *P*<0.05. For this program, white participants (75%) were significantly more likely to complete posttreatment questionnaires than participants from an ethnically minoritized background (55%), *χ*^2^ *=*4.64, *P*<0.05. Participants in the outpatient program who did and did not complete posttreatment questionnaires did not differ on any other baseline variable.

### Treatment Outcomes: Three-week Residential and Virtual High-Intensity Programs

Table 4 shows pretreatment and posttreatment scores on outcome and process variables across the in-person residential (prepandemic) and virtual high-intensity (during the pandemic) programs. For the prepandemic residential program, statistically significant improvements (all *P*s<0.001) were observed for all variables. Based on the effect sizes, these improvements were large for pain interference and depression and small for work and social adjustment, pain intensity, and pain acceptance. Pain interference and depression reduced to medium effect size improvements when analyzed using BOCF, while the effects for the other outcomes remained small when analyzed with BOCF; all of these remained statistically significant (all *P*s<0.001). On the posttreatment PGIC, 44.6% of participants reported meaningful improvement overall, while 50.8% did not report a meaningful change, and 4.6% of participants reported that they were meaningfully worse overall at the end of treatment.

**TABLE 4:** Treatment Outcomes for Virtual High-Intensity (During COVID) and In-Person Residential Programs (Pre-COVID)

Variable program	Pre mean^*^ (SE)	Post mean^*^ (SE) or frequency (%)	*t*(df) and *P*	Effect size (d)	BOCF Pre mean^*^ (SE)	BOCF Post mean^*^ (SE) or frequency (%)	BOCF *t*(df) and *P*	BOCF effect size (d)
Pain interference	—	—	−8.74 (234),	—	—	—	−8.13 (291),	—
Virtual high-intensity	7.51 (0.11)	6.68 (0.11)	*P<*0.001	0.46	7.51 (0.11)	6.89 (0.11)	*P*<0.001	0.33
Pain interference	—	—	−20.10 (514),	—	—	—	−18.5 (559),	—
Residential	7.82 (0.07)	6.38 (0.08)	*P<*0.001	0.83	7.82 (0.07)	6.60 (0.07)	*P*<0.001	0.71
Work/social adjustment	—	—	−5.81 (240),	—	—	—	−5.60 (292),	—
Virtual high-intensity	31.30 (0.43)	29.00 (0.46)	*P<*0.001	0.31	31.30 (0.43)	29.60 (0.43)	*P*<0.001	0.23
Work/social adjustment	—	—	−11.20 (509),	—	—	—	−10.70 (556),	—
Residential	32.50 (0.30)	29.10 (0.31)	*P<*0.001	0.48	32.50 (0.30)	29.60 (0.30)	*P*<0.001	0.41
Depression	—	—	−8.26 (242),	—	—	—	−7.68 (293),	—
Virtual high-intensity	16.30 (0.35)	13.40 (0.39)	*P<*0.001	0.48	16.30 (0.36)	14.10 (0.36)	*P*<0.001	0.35
Depression	—	—	−20.30 (506),	—	—	—	−18.70 (552),	—
Residential	18.10 (0.25)	13.00 (0.26)	*P<*0.001	0.87	18.1, (0.25)	13.70 (0.25)	*P*<0.001	0.73
Pain intensity	—	—	−7.34 (238),	—	—	—	−6.87 (293),	—
Virtual high-intensity	7.51 (0.09)	6.81 (0.10)	*P<*0.001	0.44	7.51 (0.10)	6.99 (0.10)	*P*<0.001	0.32
Pain intensity	—	—	−11.30 (515),	—	—	—	−10.70 (556),	—
Residential	7.80 (0.07)	6.98 (0.07)	*P<*0.001	0.49	7.80 (0.07)	7.11 (0.07)	*P*<0.001	0.42
Pain acceptance	—	—	5.87 (245),	—	—	—	5.74 (288),	—
Virtual high-intensity	16.30 (0.43)	18.80 (0.46)	*P<*0.001	0.34	16.30 (0.43)	18.20 (0.42)	*P*<0.001	0.27
Pain acceptance	—	—	10.30 (501),	—	—	—	9.67 (548),	—
Residential	16.40 (0.35)	20.10 (0.37)	*P<*0.001	0.46	16.40 (0.36)	19.50 (0.35)	*P*<0.001	0.38
PGIC: virtual high-intensity (n=225)								
Meaningful improvement	—	79 (35.1)	—	—	—	—	—	—
No meaningful change	—	137 (60.8)	—	—	—	—	—	—
Meaningful worsening	—	9 (4.0)	—	—	—	—	—	—
PGIC residential (n=480)								
Meaningful improvement	—	214 (44.6)	—	—	—	—	—	—
No meaningful change	—	244 (50.8)	—	—	—	—	—	—
Meaningful worsening	—	22 (4.6)	—	—	—	—	—	—

Effects sizes (Cohen’s *d*) interpreted as 0.20=small, 0.50=medium, 0.80=large.

* Estimated marginal mean.

BOCF indicates Imputation using baseline observation carried forward; PGIC, Patient Global Impression of Change rating.

For the virtual high-intensity program during the pandemic, statistically significant improvements (all *P*s*<*0.001) with small effect sizes were also observed for all variables from pretreatment to posttreatment. A similar pattern of results was observed when analyzed using BOCF. At the end of treatment, 35.1% of participants rated themselves as meaningfully improved overall, while 60.8% reported no meaningful change. Four percent rated themselves as meaningfully worse at posttreatment.

### Treatment Outcomes: Outpatient and Virtual Low-Intensity Programs

For the in-person outpatient program, statistically significant improvements (all *P*s<0.01) were observed from pretreatment to posttreatment for pain interference, depression, pain intensity, and pain acceptance, with small effects. When analyzed with BOCF, pain interference and depression showed small effects, while pain intensity and pain acceptance reduced to less than small effects, although these remained statistically significant (all *P*s≤0.01). There was no significant change in work and social adjustment, and the effect size was less than small across analyses with and without BOCF. On the posttreatment PGIC, 20.2% of participants reported meaningful improvement, 76.2% reported no meaningful change, and 3.6% reported that they were meaningfully worse overall.

There were no statistically significant changes on any variable for the virtual low-intensity program (*P* values ranged between 0.06 and 0.59). Although not statistically significant, a small effect size improvement was observed for pain interference, although it reduced to less than small in the analysis with BOCF. The effects for all other variables were less than small across analyses with and without BOCF. At the end of treatment, 21.8% of participants rated themselves as meaningfully improved overall and 78.2% reported no meaningful change. No participant rated themselves as meaningfully worse on the PGIC (Table 5).

**TABLE 5:** Treatment Outcomes for Virtual Low-Intensity (During COVID) and In-Person Outpatient Programs (Pre-COVID)

Variable program	Pre mean^*^ (SE)	Post mean^*^ (SE) or frequency (%)	*t*(df) and *p*	Effect size (d)	BOCF Pre mean^*^ (SE)	BOCF Post mean^*^ (SE) or frequency (%)	BOCF *t*(df) and *p*	BOCF effect size (d)
Pain interference	—	—	−1.93 (65.3),	—	—	—	−1.64 (88.2),	
Virtual low-intensity	6.44 (0.20)	6.01 (0.24)	*P*=0.06	0.22	6.44 (0.20)	6.21 (0.20)	*P*=0.10	0.12
Pain interference	—	—	−4.55 (91.5),	—	—	—	−4.04 (121),	
Outpatient	6.38 (0.17)	5.80 (0.18)	*P*<0.001	0.30	6.38 (0.17)	6.00 (0.17)	*P*<0.001	0.20
Work/social adjustment	—	—	0.85 (57.5),	—	—	—	1.13 (89),	—
Virtual low-intensity	24.20 (0.99)	24.80 (1.09)	*P*=0.40	0.07	24.20 (0.99)	24.70 (0.99)	*P*=0.26	0.06
Work/social adjustment	—	—	−1.73 (89.7),	—	—	—	−1.58 (120),	—
Outpatient	25.10 (0.77)	24.20 (0.81)	*P*=0.09	0.10	25.10 (0.77)	24.50 (0.76)	*P*=0.12	0.07
Depression	—	—	0.55 (59.1),	—	—	—	0.45 (89),	—
Virtual low-intensity	13.10 (0.62)	13.40 (0.73)	*P*=0.59	0.11	13.10 (0.63)	13.30 (0.63)	*P*=0.65	0.03
Depression	—	—	−4.27 (88.9),	—	—	—	−3.90 (121),	—
Outpatient	13.00 (0.55)	11.20 (0.59)	*P*<0.001	0.32	13.00 (0.56)	11.80 (0.55)	*P*<0.001	0.20
Pain intensity	—	—	−0.55 (75.7),	—	—	—	0.24 (89),	0.10
Virtual low-intensity	6.81 (0.20)	6.66 (0.25)	*P*=0.58	0.08	6.81 (0.20)	6.86 (0.20)	*P*=0.81	0.10
Pain intensity	—	—	−2.83 (92.6),	—	—	—	−2.50 (121),	—
Outpatient	6.87 (0.17)	6.48 (0.19)	*P*<0.01	0.20	6.87 (0.17)	6.62 (0.17)	*P*=0.01	0.13
Pain acceptance	—	—	0.81 (60.5),	—	—	—	0.57 (87),	—
Virtual low-intensity	21.80 (0.78)	22.40 (0.90)	*P*=0.42	0.12	21.80 (0.78)	22.00 (0.78)	*P*=0.57	0.04
Pain acceptance	—	—	3.47 (87.4),	—	—	—	3.21 (120),	—
Outpatient	22.60 (0.69)	24.50 (0.74)	*P*<0.001	0.26	22.6 (0.69)	23.90 (0.68)	*P*<0.01	0.17
PGIC: virtual low-intensity (n=55)								
Meaningful improvement	—	12 (21.8)	—	—	—	—	—	—
No meaningful change	—	43 (78.2)	—	—	—	—	—	—
Meaningful worsening	—	0 (0.0)	—	—	—	—	—	—
PGIC outpatient (n=84)								
Meaningful improvement	—	17 (20.2)	—	—	—	—	—	—
No meaningful change	—	64 (76.2)	—	—	—	—	—	—
Meaningful worsening	—	3 (3.6)	—	—	—	—	—	—

Effects sizes (Cohen’s *d*) interpreted as 0.20=small, 0.50=medium, 0.80=large.

* Estimated marginal mean.

BOCF indicates Imputation using baseline observation carried forward; PGIC, Patient Global Impression of Change rating.

### Treatment Outcomes: Two-week Residential and Virtual Pre-neuromodulation Programs

For the residential 2-week pre-neuromodulation program, statistically significant improvements (all *P*s<0.01) were observed from pretreatment to posttreatment for all variables. The improvements were medium for pain interference, small for work and social adjustment, depression, and pain acceptance, and less than small for pain intensity. The pattern of results was similar when analyzed with BOCF. On the PGIC, 31.1% of participants reported meaningful improvement, 64.2% reported no meaningful change, and 4.7% reported that they were meaningfully worse overall.

For the virtual pre-neuromodulation program, there were statistically significant improvements (all *P*s<0.01) with small effect sizes for pain interference, depression, and pain acceptance from pretreatment to posttreatment. Work and social adjustment (*P*=0.12) and pain intensity (*P*=0.16) did not significantly improve, and less than small effects were observed. The BOCF analyses showed a similar pattern of results. At posttreatment, 16.4% of participants rated themselves as meaningfully improved and 75.0% reported no meaningful change, while 8.6% rated themselves as meaningfully worse overall on the PGIC (Table 6).

**TABLE 6:** Treatment Outcomes for Virtual Pre-neuromodulation (During COVID) and In-Person Two-week Residential Pre-Neuromodulation Programs (Pre-COVID)

Variable program	PreMean^*^ (SE)	PostMean^*^ (SE) or frequency (%)	*t*(df) and *P*	Effect size (d)	BOCF Premean^*^ (SE)	BOCF Postmean^*^ (SE) or frequency (%)	BOCF *t*(df) and *P*	BOCF effect size (d)
Pain interference	—	—	−6.18 (109),	—	—	—	−5.83 (128),	—
Virtual neuromodulation	7.89 (0.15)	7.11 (0.16)	*P*<0.001	0.46	7.89 (0.15)	7.27 (0.15)	*P*<0.001	0.36
Pain interference	—	—	−10.90 (194),	—	—	—	−10.70 (205),	—
Residential neuromodulation	7.60 (0.11)	6.70 (0.11)	*P*<0.001	0.56	7.60 (0.11)	6.76 (0.11)	*P*<0.001	0.51
Work/social adjustment	—	—	−1.59 (112),	—	—	—	−1.48 (128),	—
Virtual neuromodulation	31.70 (0.70)	30.70 (0.74)	*P*=0.12	0.13	31.70 (0.70)	30.90 (0.69)	*P=*0.14	0.10
Work/social adjustment	—	—	−3.83 (195),	—	—	—	−3.78 (206),	—
Residential neuromodulation	31.0 (0.44)	29.60 (0.45)	*P*<0.001	0.22	31.00 (0.44)	29.70 (0.45)	*P*<0.001	0.20
Depression	—	—	−4.22 (109),	—	—	—	−4.06 (128),	—
Virtual neuromodulation	16.60 (0.55)	14.60 (0.58)	*P*<0.001	0.32	16.60 (0.55)	15.00 (0.55)	*P*<0.001	0.25
Depression	—	—	−7.62 (198),	—	—	—	−7.52 (204),	—
Residential neuromodulation	15.70 (0.43)	12.70 (0.44)	*P*<0.001	0.49	15.70 (0.43)	12.90 (0.43)	*P*<0.001	0.46
Pain intensity	—	—	−1.42 (104),	—	—	—	−1.63 (128),	—
Virtual neuromodulation	7.83 (0.12)	7.68 (0.12)	*P*=0.16	0.12	7.83 (0.12)	7.69 (0.12)	*P*=0.11	0.10
Pain intensity	—	—	−3.09 (200),	—	—	—	−3.02 (205),	—
Residential neuromodulation	7.77 (0.10)	7.50 (0.10)	*P*<0.01	0.19	7.77 (0.10)	7.51 (0.10)	*P<*0.01	0.18
Pain acceptance	—	—	3.34 (110),	—	—	—	3.14 (127),	—
Virtual neuromodulation	14.80 (0.69)	17.00 (0.74)	*P=*0.001	0.29	14.80 (0.69)	16.50 (0.69)	*P<*0.01	0.22
Pain acceptance	—	—	4.85 (193),	—	—	—	4.81 (202),	—
Residential neuromodulation	17.80 (0.60)	20.00 (0.60)	*P*<0.001	0.26	17.80 (0.60)	19.90 (0.60)	*P<*0.001	0.27
PGIC: virtual								
Neuromodulation (n=104)	—	17 (16.4)	—	—	—	—	—	—
Meaningful improvement	—	78 (75.0)	—	—	—	—	—	—
No meaningful change	—	9 (8.6)	—	—	—	—	—	—
Meaningful worsening	—	—	—	—	—	—	—	—
PGIC residential								
Neuromodulation (n=193)	—	60 (31.1)	—	—	—	—	—	—
Meaningful improvement	—	124 (64.2)	—	—	—	—	—	—
No meaningful change	—	9 (4.7)	—	—	—	—	—	—
Meaningful worsening	—	—	—	—	—	—	—	—

Effects sizes (Cohen’s *d*) interpreted as 0.20=small, 0.50=medium, 0.80=large.

* Estimated marginal mean.

BOCF indicates Imputation using baseline observation carried forward; PGIC, Patient Global Impression of Change rating.

## DISCUSSION

This study investigated outcomes associated with the implementation of a range of virtual ACT-based PMP formats in routine care during the pandemic. Posttreatment questionnaire completion rates suggest a small reduction in treatment completion (7–14%) for virtual compared with in-person programs. High-intensity ACT-based virtual programs, including for patients awaiting neuromodulation, were associated with significant improvements in pain interference, depression, and pain acceptance. Work and social adjustment and pain intensity also improved after the virtual high-intensity program. No significant improvements were observed for the low-intensity virtual program. This pattern of results across the virtual programs was generally consistent when sensitivity analyses were conducted using BOCF imputation. Across virtual programs, 16% to 35% of participants reported meaningful improvement overall, while only a small proportion, 0% to 9%, reported meaningful worsening on the global impression of change index. Taken together, the data provide preliminary support for the potential benefits of high, but not low, intensity virtual group delivery of ACT-based PMPs. The findings can inform future research and virtual treatment developments to optimize engagement and outcomes.

For the virtual high-intensity program, significant small effects were observed for all variables. This contrasts with large effects (medium when analyzed with BOCF) for pain interference and depression for the residential program. Direct comparison between outcomes for these programs must be qualified given the observational design, different contexts of data collection, and different sample sizes. However, several plausible explanations for the relative magnitude of outcomes warrant consideration. Firstly, due to challenges with fatigue and concentration with remote delivery,^26^ the treatment hours in the virtual high-intensity program were considerably reduced from the residential program, which may have limited the magnitude of improvements. In-person programs may also provide greater opportunities to foster the therapeutic alliance and sensitivity for detecting and engaging with therapeutic processes.^18,27^ Notably, though, a previous noninferiority RCT showed that in-person and video teleconferencing delivery of ACT (individual rather than group-based) produced comparable outcomes for veterans with chronic pain.^23^

The pandemic context may have limited the magnitude of change that was possible during virtual PMPs. Rates of psychological distress increased during the pandemic, including in people with persistent pain.^46–48^ Similarly, social and physical functioning were limited for substantial periods due to lockdown restrictions and social distancing. Therefore, participants’ capacity to make further gains in mood and functioning may have been limited by this context. Indeed, a number reported significant COVID-related events and worsening in functioning and mood because of COVID-19. Research is therefore needed to understand the impact of virtual PMPs beyond the pandemic.

Participants who completed a virtual PMP in preparation for receiving neuromodulation also showed significant improvements in pain interference, depression, and pain acceptance. Across residential and virtual formats of the pre-neuromodulation program, improvements were generally small, except for a medium improvement for pain interference in the residential format. Given the unique context of the pre-neuromodulation program, including specific concerns and worries that patients may have about the procedure itself, it is important to emphasize that the outcome data from this program cannot be directly compared with the other ACT-based programs reported here. However, these results add to previous findings showing that people can improve their functioning in the presence of pain while they await an intervention to control it.^17^ Research is needed to further maximize the impacts of psychologically-informed pre-neuromodulation treatment. Research is also needed to understand the extent to which improvements in such a program contribute to improvements in poststimulation outcomes.^49^

The significant small effects observed for the in-person outpatient PMP are consistent with an RCT of 4-session group-based ACT in primary care.^16^ These data support the utility of relatively brief ACT-based treatment for some people with pain. However, not all outcomes improved with the outpatient program, and there were no significant improvements in the virtual low-intensity program. In-person and virtual treatment completion was relatively low for this group of patients, which further limited the sample size and made interpretation of the effect estimates challenging. Most participants on the in-person outpatient and virtual low-intensity PMPs were in some form of employment. Therefore, difficulties fitting treatment around work may partially account for lower treatment completion.^50^ During the implementation of the virtual low-intensity program, patients suggested the need to increase the overall treatment duration to reduce the frequency of sessions per week to facilitate their attendance alongside work, and this informed treatment refinements. There is evidence that cognitive-behavioral interventions targeting the person in pain and their employer improve health care utilization and work absence.^51^ Therefore, further collaboration with employers may be needed as part of the low-intensity treatment package, given the relevance of employment-related issues for this cohort.

Another challenge of low-intensity ACT-based PMPs is how to optimally target psychological flexibility processes within a relatively brief timeframe. Considering the relatively higher level of functioning of this group, this research indicates that some people with pain remain engaged in activities while being unwilling to experience pain is of relevance.^52,53^ This may reflect a tendency to distract from pain by ‘keeping busy,’ which may come with costs in terms of increased distress.^52,53^ Supporting people to acknowledge and make space for pain and related difficulties poses a challenge in a time-limited treatment, particularly when this requires practice with slowing down their approach to activities and movement rather than ‘pushing through.’ This may be especially challenging in a virtual context where people remain in their usual environment for treatment. Therefore, research is needed to understand how psychological flexibility processes can best be targeted within this context.

If future research provides additional support for virtual PMPs, ongoing implementation of in-person and virtual PMP delivery options can increase inclusivity. Beyond the pandemic, virtual treatments have advantages for enabling patients from wider geographic areas to participate and potentially reduce costs.^18,54^ However, remote delivery is not accessible to all, including those without Internet, sufficient digital literacy, or an appropriate home environment.^55–57^ Therefore, despite the proliferation of remotely-delivered PMPs during the pandemic, in-person delivery remains important. Where services rely heavily on remote delivery, it is important to consider strategies to widen accessibility, such as loaning required technology and up-skilling patients to use this.

With the range of in-person and virtual PMPs now available, a key challenge is to match patients with the most appropriate treatment option. Incorporating patient preferences into the treatment recommendation process may have motivated engagement with treatment programs in the current study, although the impact of this is difficult to ascertain within the current observational design.^58,59^ Further understanding of predictors of treatment outcomes by delivery format may enhance our ability to match patients with the most suitable treatment format. To date, research on cognitive-behavioral pain management approaches, including ACT, has struggled to identify consistent predictors of treatment outcomes.^14,60,61^ The reliance on aggregate group-level data may contribute to the difficulty in identifying predictors, as treatment response is likely to be highly individual. Idiographic methods, such as single-case experimental designs, may facilitate a more nuanced understanding of treatment predictors and outcomes for in-person and virtual formats.^62,63^ An idiographic focus may ultimately enable researchers and treatment providers to better match patients with the most appropriate treatment format.

This study had several limitations. This was not an RCT, which limits conclusions about the causal impact of the treatments. In addition, the in-person and virtual programs cannot be unequivocally compared, given the study design and differing contexts of data collection. Experimental designs are needed to directly compare the delivery formats examined. Furthermore, the sample sizes differed considerably, which limits the interpretation of the outcomes across the treatment programs. In particular, the sample for the virtual low-intensity program was relatively small, reducing power and limiting certainty around the reported effects. Relatedly, the duration of the virtual low-intensity program was increased in response to feedback during initial implementation. Anecdotally, patients indicated that the 20-hour version was more acceptable than the 12.5-hour version. However, due to the small number of patients receiving these different versions, a comparison was not made, and these data were combined. The programs were delivered by clinicians in one specialty center and replication across other centers is needed. Finally, although there was better representation of people from ethnically minoritized backgrounds in the virtual high (34%) and low-intensity (33%) programs, participants completing the virtual pre-neuromodulation program were predominantly white (92%). Therefore, the lack of generalizability to ethnically minoritized participants is a key limitation of data from the pre-neuromodulation programs. To mitigate the risk of perpetuating inequities,^64^ research is needed to understand the barriers to ethnically minoritized patients being referred for neuromodulation and this form of preparatory treatment.

Despite these limitations, this study provides preliminary support for the potential benefits of higher-intensity virtual PMPs, although research is needed to maximize treatment engagement and outcomes. The availability of virtual programs in addition to in-person options, can facilitate greater inclusivity of services to meet the needs of a broad spectrum of patients. A key challenge moving forward is to match patients with the delivery format that best meets their needs.

## Supplementary Material

SUPPLEMENTARY MATERIAL
