# Supplementary material for: An Observational Study of Outcomes Associated With Virtual Pain Management Programs Based on Acceptance and Commitment Therapy Implemented During the COVID-19 Pandemic
Source: Clin J Pain. 2023 Jul 4;39(10):524–36. doi: 10.1097/AJP.0000000000001144 (PMC10498868; doi:10.1097/AJP.0000000000001144)
Supplement: SUPPLEMENTARY MATERIAL [file ajp-39-524-s001.docx]

| Table S1. COVID-19 events experienced and impact. | **Virtual High Intensity**  M(SD) or *n*(%)  Pre-treatment  *n=* 294 | **Virtual Low Intensity**  M(SD) or *n*(%)  Pre-treatment  *n=* 90 | **Virtual Pre-**  **NM**  M(SD) or *n*(%)  Pre-treatment  *n=* 129 |
| --- | --- | --- | --- |
| **COVID-19 Events (not mutually exclusive)**  I have been ill with COVID  Caring for a friend/flatmate with COVID  Caring for family/partner with COVID  Death of spouse/partner  Separation/divorce  Being fired/lost job  Death of a friend/family member  Major financial change (better)  Major financial change (worse)  Major change in living conditions  None of the above | 59 (20.5)  1 (0.3)  12 (4.2)  1 (0.3)  3 (1.0)  20 (6.9)  45 (15.6)  4 (1.4)  52 (18.1)  49 (17.0)  149 (51.7) | 17 (19.5)  0 (0)  4 (4.6)  0 (0)  1 (1.1)  4 (4.5)  10 (11.4)  1 (1.1)  13 (14.9)  9 (10.3)  52 (59.1) | 15 (11.7)  0 (0)  2 (1.6)  0 (0)  2 (1.6)  6 (4.7)  9 (7.0)  0 (0)  21 (16.4)  8 (6.3)  84 (65.6) |
| **Change in healthcare use**  Reduced  No change  Increased  Missing | 155 (52.8)  102 (34.6)  30 (10.2)  7 (2.4) | 37 (41.1)  47 (52.3)  4 (4.4)  2 (2.2) | 63 (48.9)  56 (43.5)  9 (7.0)  1 (0.8) |
| **Change in health/functioning overall**  Meaningful Improvement  No Meaningful Change  Meaningful Worsening  Missing | 13 (4.4)  153 (52.0)  121 (41.2)  7 (2.4) | 5 (5.5)  58 (64.5)  24 (26.7)  3 (3.3) | 2 (1.6)  90 (69.8)  36 (27.9)  1 (0.8) |
| **Change in physical activities**  Meaningful Improvement  No Meaningful Change  Meaningful Worsening  Missing | 10 (3.4)  144 (49.0)  133 (45.2)  7 (2.4) | 5 (5.5)  45 (50.0)  38 (42.2)  2 (2.2) | 1 (0.8)  78 (60.4)  49 (38.0)  1 (0.8) |
| **Change in social activities**  Meaningful Improvement  No Meaningful Change  Meaningful Worsening  Missing | 4 (1.3)  118 (40.2)  165 (56.2)  7 (2.4) | 1 (1.1)  45 (50.0)  42 (46.6)  2 (2.2) | 1 (0.8)  65 (50.4)  62 (48.1)  1 (0.8) |
| **Change in work-related activities**  Meaningful Improvement  No Meaningful Change  Meaningful Worsening  Missing | 6 (2.0)  162 (55.2)  119 (40.5)  7 (2.4) | 1 (1.1)  60 (66.7)  26 (28.9)  3 (3.3) | 2 (1.6)  84 (65.2)  42 (32.6)  1 (0.8) |
| **Change in mood**  Meaningful Improvement  No Meaningful Change  Meaningful Worsening  Missing | 13 (4.4)  155 (52.7)  119 (40.5)  7 (2.4) | 3 (3.3)  56 (62.2)  29 (32.2)  2 (2.2) | 1 (0.8)  78 (60.5)  49 (38.0)  1 (0.8) |
| **Change in pain**  Meaningful Improvement  No Meaningful Change  Meaningful Worsening  Missing | 4 (1.4)  128 (43.6)  155 (52.7)  7 (2.4) | 4 (4.4)  49 (54.4)  34 (37.8)  3 (3.3) | 2 (1.6)  55 (58.9)  50 (38.7)  1 (0.8) |
| **Social support satisfaction**  Meaningful Improvement  No Meaningful Change  Meaningful Worsening  Missing | 55 (18.7)  168 (57.1)  64 (21.7)  7 (2.4) | 10 (11.2)  69 (76.6)  8 (8.9)  3 (3.3) | 17 (13.2)  94 (72.9)  17 (13.2)  1 (0.8) |

Note: NM, Pre-neuromodulation

*^1^* Percentages may not sum 100% because of rounding.

^2^ Anchors:

Meaningful improvement = 1- very much improved, 2- much improved

No meaningful change = 3- minimally improved, 4- no change, 5- minimally worse

Meaningful worsening = 6- much worse, 7- very much worse.
